# Can photobiomodulation prevent herpes labialis recurrences? A systematic review

**DOI:** 10.31744/einstein_journal/2026RW2359

**Published:** 2026-09-01

**Authors:** Karen Müller Ramalho, Nicolly Dias dos Santos, Juliana Mota Siqueira, Anna Carolina Ratto Tempestini Horliana, Carlos de Paula Eduardo, Leticia Mello Bezinelli, Fernanda de Paula Eduardo, Anna Luíza Damaceno Araújo

**Affiliations:** 1 Faculdade Israelita de Ciências da Saúde Albert Einstein Hospital Israelita Albert Einstein São Paulo SP Brazil Faculdade Israelita de Ciências da Saúde Albert Einstein, Hospital Israelita Albert Einstein, São Paulo, SP, Brazil.; 2 Universidade Nove de Julho São Paulo SP Brazil Universidade Nove de Julho, São Paulo, SP, Brazil.; 3 Universidade de São Paulo São Paulo SP Brazil Universidade de São Paulo, São Paulo, SP, Brazil.

**Keywords:** Herpes simplex, Herpes labialis, Lasers, semiconductor, Low-level light therapy, Photobiomodulation, Recurrence

## Abstract

**Objective:**

This systematic review evaluated the effectiveness of photobiomodulation for preventing recurrent herpes labialis.

**Methods:**

Eligibility criteria were defined using the PICOS framework, and only randomized clinical trials were included. We searched PubMed, Embase, Scopus, the Cochrane Library, the Virtual Health Library, and gray-literature sources. Of the records identified, nine reports underwent full-text assessment, and three studies met the eligibility criteria.

**Results:**

Two trials reported lower recurrence frequencies after photobiomodulation compared to control interventions, while one trial found no difference. Findings regarding recurrence severity and duration were inconsistent across studies. No adverse events were reported in the studies that provided safety data.

**Conclusion:**

The available evidence suggests that photobiomodulation may reduce the recurrence frequency, severity, and duration of herpes labialis. However, the certainty of the evidence was rated as low due to small sample sizes, incomplete blinding, and variations in laser parameters and comparators. These limitations restrict generalizability and preclude definitive clinical recommendations. Well-designed randomized controlled trials with standardized protocols and consistent outcome measures are needed to establish the clinical utility of photobiomodulation for recurrent herpes labialis.

## INTRODUCTION

Herpes simplex virus (HSV) infection is highly prevalent worldwide, with HSV-1 and HSV-2 affecting billions of people.^(1)^ According to the World Health Organization, approximately 3.7 billion people younger than 50 years, representing 67% of the global population, have HSV-1 infection.^(2)^ Herpes simplex virus-1 is transmitted primarily through oral contact and commonly causes recurrent disease. Herpes simplex virus reactivation may be triggered by stress, immunosuppression, and ultraviolet-light exposure.^(1,3)^ Recurrent herpes labialis can impair quality of life by causing embarrassment, social withdrawal, anxiety, and reduced self-esteem, particularly among individuals with frequent or unpredictable outbreaks.^(4)^

Despite decades of research, no effective preventive or therapeutic HSV vaccine is available. Limited understanding of virus–host interactions and the insufficient efficacy of candidate vaccines in clinical trials have hindered vaccine development.^(5)^ Oral antiviral agents, including acyclovir and valacyclovir, are commonly used to manage HSV infection and may reduce outbreak duration and severity. However, their preventive effectiveness is limited. A Cochrane review reported inconsistent findings for short-term antiviral therapy. Long-term suppressive treatment may reduce recurrence frequency, but the clinical benefit appears modest and is supported by low-certainty evidence.^(6)^ The review found no evidence of an increased risk of adverse events with long-term therapy; however, treatment decisions should consider adherence, cost, potential adverse effects, and antiviral resistance.^(6)^ Photobiomodulation using low-power laser irradiation has been investigated as an alternative strategy for preventing recurrent herpes labialis. When applied during the early stages of an active herpes labialis outbreak, photobiomodulation may reduce pain, inflammation, and healing time through effects on cellular repair and local immune responses.^4,7^ Recent studies have reported potentially favorable effects of photobiomodulation in both the treatment of active lesions and the prevention of recurrent herpes labialis.^5,9^ Repeated photobiomodulation sessions may also reduce the frequency of future outbreaks in patients with recurrent herpes labialis. Photobiomodulation has been proposed as a noninvasive adjunct to conventional antiviral therapy.^4,10^

Although antiviral agents are commonly prescribed to reduce herpes labialis recurrences, their preventive benefit is modest, and no preventive treatment is currently considered the standard of care.^(6)^ In this context, photobiomodulation has emerged as a potential adjunctive strategy because it may modulate inflammation, promote tissue repair, and influence cellular responses associated with HSV reactivation. However, most published studies have examined photobiomodulation for active herpes labialis lesions, whereas its use for recurrence prevention remains insufficiently studied. A systematic evaluation of the evidence on preventive photobiomodulation protocols is therefore warranted.

By analyzing randomized controlled trials, this systematic review examined whether photobiomodulation reduces recurrence frequency, severity and duration.

## OBJECTIVE

The review also aimed to determine the certainty of evidence, identify gaps in the published literature, and inform clinical practice and future research.

## METHODS

### Eligibility criteria

The review question and eligibility criteria were defined using the PICOS (Table 1) framework: “Is photobiomodulation effective for preventing herpes labialis recurrences?” The population comprised patients with recurrent herpes labialis; the intervention was preventive photobiomodulation or low-level laser therapy; the outcomes included recurrence frequency and recurrence severity; and the eligible study design was a randomized controlled trial. Restricting eligibility to randomized controlled trials was intended to reduce placebo effects, observer bias, and confounding, particularly for outcomes that relied partly on participant reporting, such as recurrence frequency, symptom severity, and lesion duration. No restrictions were applied to participant characteristics, photobiomodulation parameters, or minimum follow-up duration. Studies that exclusively evaluated treatment of active herpes labialis lesions and case series were excluded.

**Table 1:** PICOS framework

PICOS component	Search terms
Population	herpes OR “herpes simplex” OR “herpes labialis” OR HSV
Intervention	photobiomodulation OR “low-level laser therapy”
Comparator	No restriction applied
Outcome	prevention of herpes labialis recurrences

HSV: herpes simplex virus; PICOS: population, intervention, comparator, outcome, and study design.

### Protocol and registration

This systematic review was conducted in accordance with the Preferred Reporting Items for Systematic Reviews and Meta-Analyses (PRISMA) 2020 statement.

### Information sources and search strategy

Database-specific searches were conducted on July 2026, in PubMed, Embase, Scopus, the Cochrane Library, and the Regional Portal of the Virtual Health Library. Gray literature was searched through Google Scholar and ProQuest. The reference lists of the included articles and relevant systematic reviews were manually screened to identify additional records not retrieved through the database searches. No restrictions were applied by language or publication date. The complete search strategies are provided in Table 1S Supplementary Material, Supplementary Material. Materials related to the searches, study selection, and data extraction are available from the corresponding author upon reasonable request.

### Selection process

Duplicate records were identified in Rayyan and removed after manual verification.^(13)^ After duplicate removal, two reviewers independently screened the titles and abstracts of all retrieved records. Reports considered potentially eligible were retrieved in full text and assessed against the predefined eligibility criteria. Disagreements were resolved through discussion and, when necessary, consultation with a third reviewer.

### Data collection process and data items

One reviewer extracted the data, and two additional reviewers independently verified the extracted information. Data extraction was guided by a customized form based on recommendations from the Cochrane Collaboration. Qualitative and quantitative data were tabulated in Microsoft Excel^®^. Extracted variables included the author and publication year; country; sample size by group and overall; baseline comparability; sample-size calculation; participant age and sex; study design; randomization and allocation methods; intervention and comparator groups; eligibility criteria; blinding of treatment and outcome assessment; attrition; study limitations reported by the authors; laser-device specifications; irradiation parameters; treatment schedule; follow-up duration; recurrence-assessment methods; recurrence frequency, severity, duration, and pain; adverse events; management of recurrences occurring during the preventive protocol; and study conclusions. Laser specifications included wavelength, operating mode, contact mode, application technique, energy density, energy per point, total energy, irradiated area, number and anatomical location of application points, spot size, placebo procedures, power output, and verification of power emission.

### Risk-of-bias assessment

Two reviewers independently assessed the risk of bias in the included randomized controlled trials using the Cochrane Risk of Bias 2 tool.^(14)^

### Effect measures

Effectiveness was evaluated using recurrence frequency, expressed as the number of herpes labialis episodes per month or year, and recurrence severity, including lesion size, edema, vesicular-phase duration, healing time, and pain.

### Synthesis methods

A narrative synthesis was conducted to integrate the findings of the included studies. A meta-analysis was not performed because the studies were not sufficiently comparable for quantitative pooling. Binary recurrence outcomes at fixed time points, such as recurrence within 6 months, were reported inconsistently, and follow-up intervals were not standardized across studies. Furthermore, not all studies reported time-to-event estimates, such as hazard ratios with 95% confidence intervals, further limiting quantitative synthesis.

### Certainty assessment

The certainty of the body of evidence for the prespecified outcomes was assessed using the GRADE approach.^(15,16)^The assessment was conducted using GRADEpro and considered risk of bias, inconsistency, indirectness, imprecision, publication bias, and other relevant factors. Certainty was classified as high, moderate, low, or very low for each outcome.

## RESULTS

### Study selection

The searches identified a total of 938 records (140 for grey literature and 798 for databases search). After screening and full-text assessment, three randomized clinical trials met the eligibility criteria and were included in the review.^10^ The study-selection process is summarized in the PRISMA flow diagram (Figure 1). Reasons for excluding reports after full-text assessment are provided in Table 2S, Supplementary Material.

**Figure 1:**
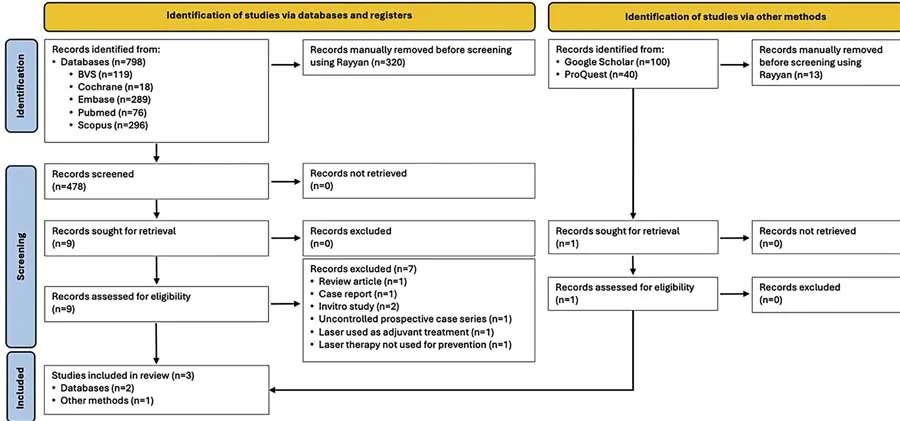
PRISMA 2020 flow diagram of study identification and selection through databases and other sources

### Study characteristics

Detailed characteristics of the included studies are presented in table 2, intervention and follow-up protocols in table 3, and clinical outcomes in table 4*.*

**Table 2:** Study characteristics and participant demographics

Characteristic	Schindl et al., 1999^(10)^	Carvalho et al., 2010^(^^12^^)^	Zanella et al., 2022^(^^11^^)^
Country	Austria	Brazil	Brazil
Number of participants by group	Laser group: n=25 Placebo Group: n=25	Laser group: n=41 Control group: n=30; topical 5% acyclovir five times daily	Laser 1 group: n=61 Laser 2 group: n=50 Placebo Group: n=47
Total participants	50	71	158
Baseline comparability	Groups were compared by age, sex, and infection site. Participants had at least one herpes outbreak per month for more than 6 months.	No demographic table was provided. Participants were monitored for 8 months before treatment, and the groups were matched by baseline recurrence frequency (0.32 episodes/month).	Groups were compared by age, sex, alcohol use, smoking status, the effect of recurrent herpes labialis on quality of life, and episode-related pain.
A priori sample-size calculation	NR	NR	NR
Participant age, years	Laser group: 31.3±12.1 Placebo Group: 36.6±14.8	28.8 [confirm summary statistic and dispersion]	Laser 1 group: 34.7 (30.0–42.1) Laser 2 group: 31.9 (27.1–40.1) Placebo Group: 34.7 (30.0–40.3)
Sex, male/female	Laser group: 5/19 Placebo Group: 6/18	All groups: 20/51	Laser 1 group: 45/16 Laser 2 group: 35/15 Placebo Group: 36/11
Study design	Randomized, double-blind, placebo-controlled trial	Randomized clinical trial	Randomized, single-blind, placebo-controlled trial
Randomized	Yes	Yes	Yes
Random-sequence generation	NR	NR	NR
Allocation concealment	NR	NR	NR
Study groups	Laser group (n=24 analyzed) Placebo Group (n=24 analyzed)	Laser group (n=41) Topical 5% acyclovir group (n=30)	Laser 1 group, 1J per point (n=61) Laser 2 group, 2J per point (n=50) Placebo Group (n=47)
Inclusion criteria	At least one herpes outbreak per month for more than 6 months	Self-reported recurrent herpes labialis for at least 3 consecutive years	Age >18 years and self-reported recurrent herpes labialis
Exclusion criteria	Current antiviral or immunosuppressive therapy, homeopathy, acupuncture, or HIV infection	Previous laser phototherapy or systemic acyclovir treatment, herpes zoster, or symptoms of an active herpes labialis outbreak	Pregnancy or breastfeeding, skin disease, recent medication use [confirm intended meaning], or antiviral-agent use
Treatment bias	All participants were treated by the same physician.	All participants were treated by the same dentist.	All participants were treated by the same dentist.
Outcome-assessor blinding	Yes; the evaluator was unaware of the study protocol.	No; the evaluator was not blinded.	No; the evaluator was not blinded.
Participant attrition	n=2, with one dropout per group	NR	NR
Limitations reported by the study authors	Pre-existing medications were continued unchanged during the trial. sample size	Sample size	Use of participant-reported questionnaires and limited follow-up duration

Data are presented as mean±standard deviation, median (interquartile range), or n, as reported by the original studies. Abbreviations: NR, not reported; RHL, recurrent herpes labialis.

**Table 3:** Photobiomodulation protocols, irradiation parameters, and follow-up assessments

Parameter	Schindl et al., 1999^(^^10^^)^	Carvalho et al., 2010^(^^12^^)^	Zanella et al., 2022^(^^11^^)^
Laser device and manufacturer	Diode laser; Helbo Lasers, Austria	Twin Laser diode laser; MMO, Brazil	Therapy EC diode laser; DMC, Brazil [confirm manufacturer]
Wavelength	690nm	780nm	808nm
Power output	80mW	60mW	100mW
Verification of power output	NR	NR	Performed
Operating mode	Continuous wave	Continuous wave	Continuous wave
Contact mode	NR	Contact	Contact
Irradiation technique	NR	Point application	Point application
Number of irradiation points	Not reported; irradiation was described only as being applied “at the site of the original chronic herpes infection.”	60 points, with 30 points per lip	Approximately 40–50 points
Irradiation time	10 min (600 s) per treated site [confirm unit of application]	2 s per point; 120 s total 3 s per point; 180 s total	Laser 1: 10 s per point; approximately 400–500 s total Laser 2: 20 s per point; approximately 800–1000 s total
Energy density,J/cm^2^	48J/cm^2^	3J/cm^2^ during preventive treatment; 4.5J/cm^2^ for active recurrences	11.11J/cm^2^ in Laser 1; 22.22J/cm^2^ in Laser 2
Energy delivered per point	Not reported	Not reported; calculated by the review authors as 0.12J per point during preventive treatment and 0.18J per point during treatment of active lesions using E (J) = P (W) × t (s).	Laser 1: 1J per point; Laser 2: 2J per point
Total energy	48J [confirm whether per point,per treated site, or per session]	7.2J during preventive treatment; 10.8J during treatment of active lesions	NR
Irradiated area, cm^2^	1 cm^2^	NR	
Anatomical irradiation site	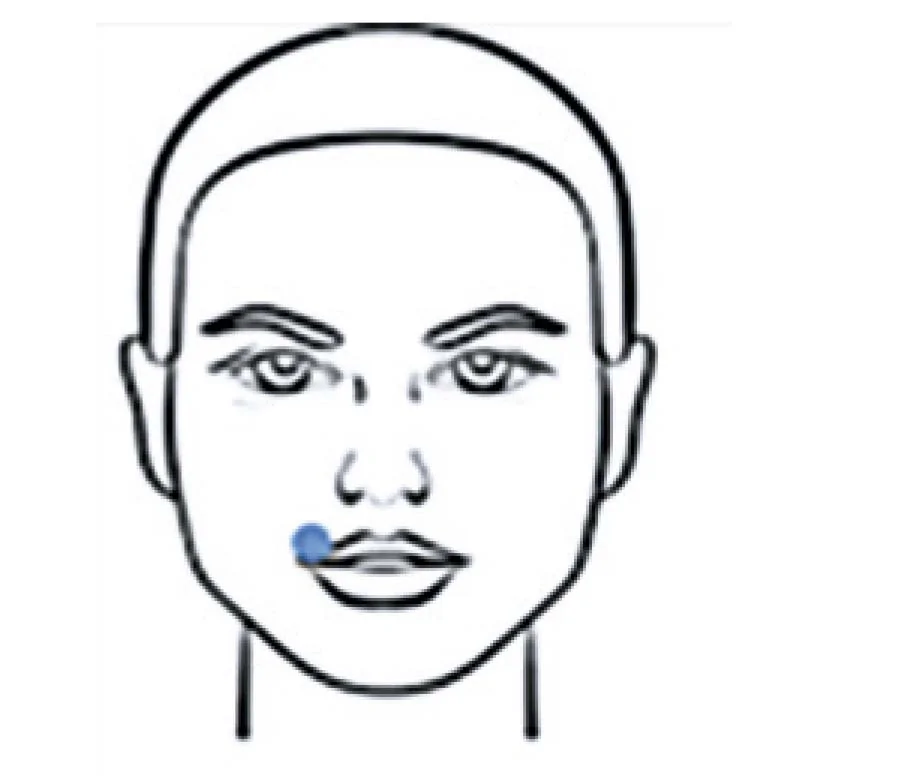	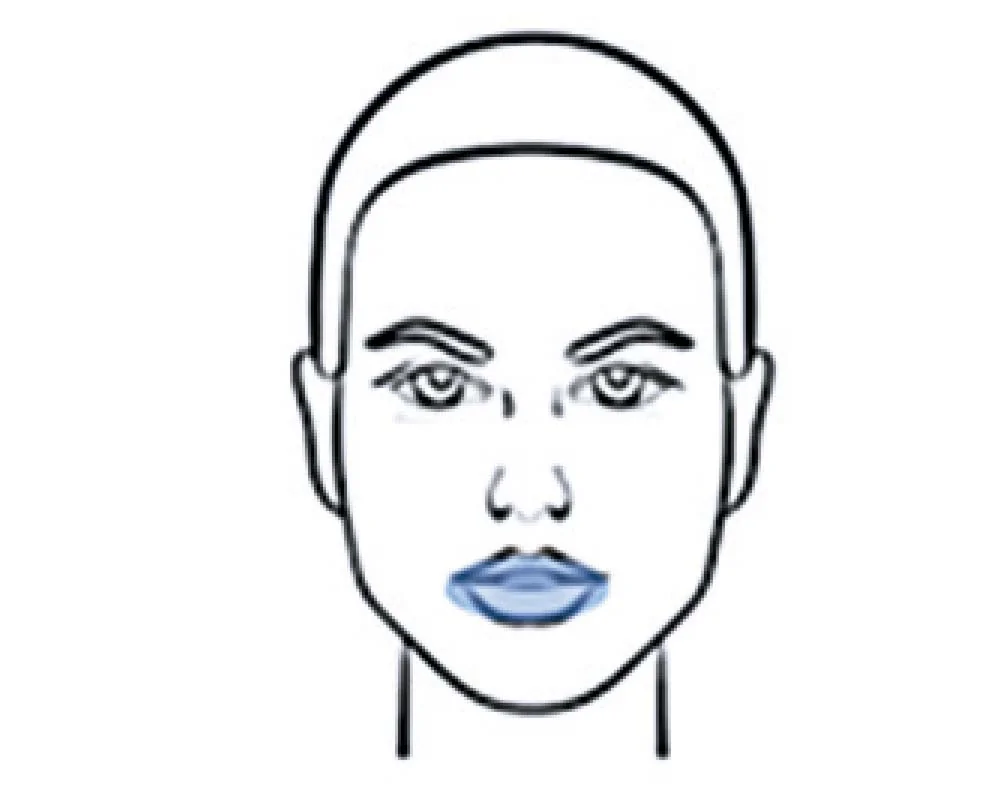	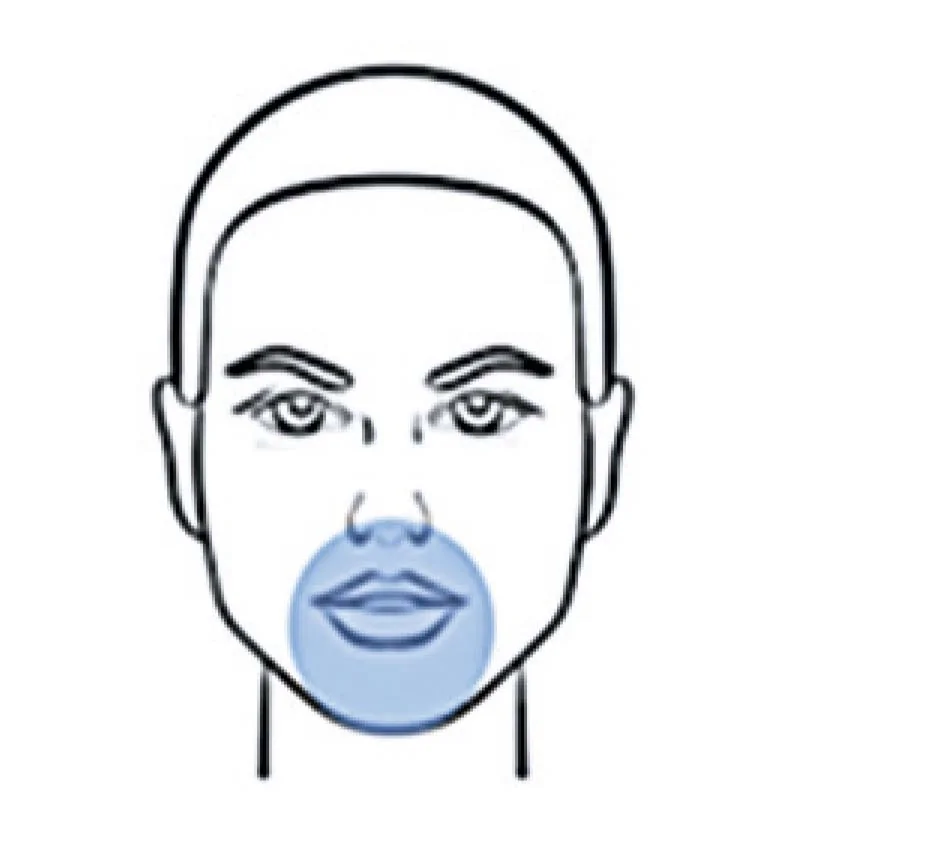
Spot area, cm^2^	NR	0.04 cm^2^	0.09 cm^2^
Total number of sessions	10	10	Phase 1: 10 sessions; phase 2: 5 sessions
Interval between sessions	Daily sessions*	Once weekly	Every 48 h
Total treatment duration	Approximately 2 weeks	10 weeks	Not reported;
Follow-up duration		52 weeks (approximately 12 months) 64 weeks (approximately 16 months) 2 years	
Method and frequency of recurrence assessment	Participants were instructed to attend the clinic when a recurrence occurred.	Weekly clinical interview; the mode of contact was not reported.	Participants were assessed using questionnaires.
Sham-irradiation procedure	Sham irradiation followed the active-treatment procedure, but the laser was switched off.	Not applicable; topical acyclovir was used as the comparator.	Sham irradiation followed the active-treatment procedure, but the laser was switched off.

Values identified as calculated were derived by the review authors and were not explicitly reported in the primary studies. The original Schindl report did not specify whether weekend treatments were administered, so exclusion of weekends should not be presented as confirmed. Abbreviations: J, joule;mW, milliwatt;nm, nanometer; NR, not reported; PBM, photobiomodulation; s, second; W, watt. *The original report did not specify whether treatment was administered on weekends; the review authors inferred that weekends were excluded.

**Table 4:** Clinical outcomes of photobiomodulation for preventing recurrent herpes labialis

Outcome	Schindl et al., 1999^(10)^	Carvalho et al., 2010^(12)^	Zanella et al., 2022^(11)^
Recurrence frequency	Significant between-group difference: p<0.0001 Laser group: median recurrence-free interval, 37.5 weeks (range, 2-52 weeks; 95% CI, 24-42 weeks) Placebo Group: median recurrence-free interval, 3 weeks (range, 1-20 weeks; 95% CI, 2-4 weeks)	No significant between-group difference: p=0.076 Laser group: 0.076 recurrences/month Control Group: 0.116 recurrences/month	Significant between-group differences were reported at 6 months (p=0.007) and 2 years (p=0.047). No significant between-group difference was observed at the 1-year assessment (p=0.071). First assessment, 6 months: Number of events (25-75%) Laser 1: 1.0 (0.0-2.0) Laser 2: 1.0 (0.0-2.0) Placebo: 3.0 (1.0-4.0) Second assessment, 1 year: Number of events (25-75%) Laser 1: 0.0 (1.0 - 2.0) Laser 2: 0.0 (1.0 - 2.0) Placebo: 0.0 (2.0 - 4.0) Third assessment, 2 years: Number of events (25-75%) Laser 1: 1.0 (0.0-1.0) Laser 2: 1.0 (0.0-2.7) Placebo: 2.0 (1.0-5.0)
Participant-reported lesion size	Not evaluated	Significant between-group difference: p=0.013 Laser group: mean score, 0.122 Control Group: mean score, 0.223 Scale: 0, absent; 1, small (0.1-2.0 mm); 2, medium (2.1-5.0 mm); and 3, large (>5.0 mm).	A significant between-group difference was observed only at the 1-year follow-up (p<0.001). No significant between-group differences were observed at 6 months (p=0.072) or 2 years (p=0.091). First posttreatment assessment, 6 months: n (%) Laser 1: Yes, 22 (84.6%); No, 4 (15.4%) Laser 2: Yes, 20 (90.9%); No, 2 (9.1%) Placebo: Yes, 8 (36.4%); No, 14 (63.6%) Second posttreatment assessment, 1 year: n (%) Laser 1: Yes, 21 (84.0%); No, 4 (16%) Laser 2: Yes, 18 (85.7%); No, 3 (14.3%) Placebo: Yes, 5 (31.3%); No, 11 (68.8%) Third posttreatment assessment, 2 years: n (%) Laser 1: Yes, 22 (91.7%); No, 2 (8.3%) Laser 2: Yes, 11 (68.8%); No, 5 (31.3%) Placebo: Yes, 7 (63.6%); No, 4 (36.4%)
Inflammatory edema	Not evaluated	Significant between-group difference: p=0.031 Laser group: mean score, 0.015 Control Group: mean score, 0.0196 Scale: 0, absent; 1, mild swelling; 2, moderate swelling; and 3, swelling involving 1 cm^2^.	Not evaluated
Vesicular phase duration	Not evaluated	Not evaluated	No significant between-group differences in shorter vesicular-phase duration were observed at 6 months, 1 year, or 2 years. p=0.072, p=0.098, and p=0.467, respectively. _________________________________________________________ *Data below:* First posttreatment assessment, 6 months: n (%) L1: Yes 8 (30.8%); No 18 (69.2%) L2: Yes 5 (22.7%) No 17 (77.3%) Placebo: Yes 1 (4.5%) No 21 (95.5%) P=0.072 Second posttreatment assessment, 1 year: n (%) L1: Yes 6 (24.0%) No 19 (76.0%) L2: Yes 5 (23.8%) No 16 (76.2%) Placebo: Yes 0 (0.0%). No 16 (100.0%) p=0.098 Third posttreatment assessment, 2 years: n (%) L1: Yes 3 (12.5%). No 21 (87.5%) L2: Yes 2 (12.5%). No 14 (87.5%) Placebo: Yes 0 (0.0%). No 11 (100.0%) p=0.467
Participant-reported faster healing of recurrent lesions	Not evaluated	Not evaluated	A significant between-group difference was observed only at the 6-month assessment (p=0.001). p=0.001 _______________________________________________________ *Data below:* First posttreatment assessment, 6 months: n (%) L1: Yes 20 (76.9%). No 6 (23.1%) L 2: Yes 20 (90.9%). No 2 (9.1%) Placebo: Yes 9 (40.9%). No 13 (59.1%) (p=0.001) Second posttreatment assessment, 1 year: n (%) L1: Yes 20 (80.0%). No 5 (20.0%) L2: Yes 15 (71.4%) No 6 (28.6%) Placebo: Yes, 8 (50.0%). No 8 (50.0%) (p=0.123) Third posttreatment assessment, 2 years: n (%) L1: Yes 21 (87.5%). No 3 (12.5%) L2: Yes 11 (68.8%). No 5 (31.3%) Placebo: Yes 6 (54.5%). No 5 (45.5%) (p=0.094)
Pain	Not evaluated	No significant between-group difference: p=0.051 Visual analog scale, 0-10 Laser group: mean score, 0.113 Control Group: mean score, 0.184	No significant between-group difference (p=0.102)
Adverse events	No adverse events were reported.	NR	No adverse events were reported.
Management of recurrences occurring during the preventive protocol	NR	A higher energy density of 4.5J/cm^2^ was applied during the prodromal, vesicular, or crusted stage of an active recurrence.	For vesicular lesions, photodynamic therapy using 660nm, 4 J, 40 s, 100mW, and 0.01% methylene blue was performed after drainage of the vesicle.
Authors’ conclusion	Ten daily low-intensity laser treatments reduced the incidence of local recurrent perioral herpes simplex infection.	Under the tested parameters, laser phototherapy reduced lesion size and inflammatory edema.	The laser groups had fewer annual recurrences than the Placebo Group, supporting a potential preventive effect of PBM on recurrence frequency and postirradiation lesion severity. No adverse events were reported.

Data are presented as median (interquartile range), n (%), mean score, or other summary measures as reported by the original studies. Statistical significance was defined according to the analyses reported by the study authors. Several entries are incomplete or internally inconsistent and should be checked against the primary publications before finalization.

95%CI: 95% confidence interval; J: joule; L1: Laser 1 group; L2: Laser 2 group;mW: milliwatt;nm: nanometer; NR: not reported; PBM: photobiomodulation; RHL: recurrent herpes labialis; VAS: visual analog scale.

The review included three controlled clinical trials that evaluated preventive photobiomodulation protocols: Schindl et al.,^(10)^ Carvalho et al.,^(12)^and Zanella et al.^(11)^

Eligibility criteria varied across the studies. Zanella et al.^(11)^ enrolled adults with self-reported recurrent herpes labialis. Carvalho et al.^(12)^ included patients with recurrent herpes labialis for at least 3 consecutive years, whereas Schindl et al.^(10)^ required at least one outbreak per month for more than 6 months. Only Schindl et al.^(10)^ explicitly required a high baseline recurrence frequency. Exclusion criteria also differed across studies. Zanella et al.^(11)^ excluded participants who were pregnant or breastfeeding, had skin disease or a recent history of medication use, or had used antiviral agents. Carvalho et al.^(12)^ excluded participants with previous laser phototherapy or systemic acyclovir treatment and those with herpes zoster. Carvalho et al.^(12)^ also excluded patients with any symptoms of an active outbreak. Schindl et al.^(10)^ excluded those receiving antiviral or immunosuppressive therapy, homeopathy, or acupuncture and those with HIV infection (Table 2).

All three studies were described as randomized, but none reported the method used to generate the allocation sequence. The reported sample sizes were 158, 71, and 50 participants for Schindl et al., Zanella et al., and Carvalho et al., respectively; none reported an a priori sample-size calculation.^10^Only Schindl et al.^(10)^ reported participant attrition. All studies reported some degree of baseline comparability, although the variables used to assess comparability differed across studies (Table 2). The trial by Carvalho et al.^(12)^was unblinded, that by Zanella et al.^(11)^was single-blind, and that by Schindl et al.^(10)^ was double-blind.

Zanella et al.^(11)^ and Schindl et al.^(10)^ used placebo controls, whereas Carvalho et al.^(12)^ used topical 5% acyclovir as the comparator. In both placebo-controlled studies, the laser device was switched off during sham irradiation; however, neither report stated whether operational cues, such as an activation sound, were reproduced to preserve participant blinding.

The anatomical sites of irradiation also differed. Schindl et al.^(10)^ treated the site of the original recurrent infection, Carvalho et al.^(12)^ treated the lips and surrounding labial region, and Zanella et al.^(11)^ treated the broader perioral area.

### Treatment and follow-up

Two studies used near-infrared laser irradiation,^(11,12)^whereas one used red-light irradiation.^(10)^ All studies used continuous-wave laser irradiation. Two studies reported using contact application at discrete irradiation points.^(11,12)^ Treatment frequency varied from daily irradiation^(10)^ to sessions every 48 hours^(11)^ or once weekly.^(12)^

Full irradiation parameters and treatment schedules for all three studies are presented in table 3. Laser power ranged from 60 to 100mW across the studies, and energy density ranged from 3 to 48J/cm^2^. Only Zanella et al.^(11)^ reported verifying laser power output before irradiation. All studies administered an initial course of 10 photobiomodulation sessions, and Zanella et al.^(11)^administered five additional sessions after 6 months. The reported treatment duration ranged from 2 to 10 weeks, and follow-up ranged from approximately 1 to 2 years.

The frequency and method of recurrence assessment differed across studies. In Schindl et al.,^(10)^ participants were instructed to attend the clinic when a recurrence occurred. Carvalho et al.^(12)^ conducted weekly clinical interviews, whereas Zanella et al.^(11)^ assessed recurrences using questionnaires. No adverse events were reported in two studies,^(10,11)^ whereas Carvalho et al.^(12)^ did not provide adverse-event information.

Two studies described how recurrences arising during the preventive protocol were managed. Carvalho et al.^(12)^ used a higher-energy irradiation parameter, whereas Zanella et al.^(11)^ applied photodynamic therapy to active lesions (Table 4).

### Recurrence frequency

Schindl et al.^(10)^ and Zanella et al.^(11)^ reported lower recurrence frequencies in the photobiomodulation Groups than in the placebo groups. Schindl et al.^(10)^ reported p<0.0001. Zanella et al.^(11)^ reported p=0.007 at 6 months and p=0.047 at 2 years. Carvalho et al.^(12)^ found no statistically significant difference in recurrence frequency between the photobiomodulation and Control Groups (Table 4).

### Recurrence severity

Carvalho et al.^(12)^ reported smaller recurrent lesions and less inflammatory edema in the photobiomodulation Group than in the Control Group. Zanella et al.^(11)^ reported smaller lesions in the Photobiomodulation Groups than in the Placebo Group only at the 1-year assessment, with no significant between-group differences at the other follow-up points. Zanella et al.^(11)^ reported faster lesion healing only at the 6-month assessment.

### Pain

No statistically significant reduction in pain was demonstrated.

### Adverse events

No adverse events were reported in the two studies that provided safety information.^(10,11)^

### Risk of bias in the included studies

In the RoB 2 assessment (Figure 2), concerns regarding deviations from intended interventions arose because sham irradiation was delivered with the laser switched off in the placebo-controlled studies,^(10,11)^ and Carvalho et al.^(12)^ compared two visibly different interventions without clarifying whether participants were informed about the alternative treatment. These features may have affected participant expectations and adherence to the assigned interventions. For both placebo-controlled studies, the predicted direction of bias arising from deviations from intended interventions was judged to favor the experimental intervention.

**Figure 2:**
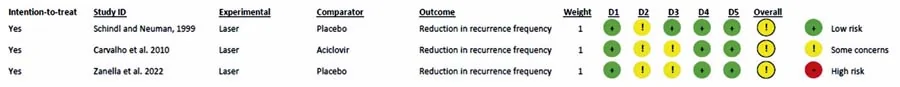
Risk of bias assessment using the ROB 2 tool for the three included studies. The table shows the judgments for each of the five domains (D1: randomization; D2: deviations from interventions; D3: missing data; D4: outcome measurement; D5: selective reporting) and the overall risk of bias judgment for each study. The symbol (+) indicates low risk in the corresponding domain

In the missing-outcome-data domain, Carvalho et al.^(12)^ did not report attrition or clearly state the final sample size in each treatment group. Similarly, Zanella et al.^(11)^ did not report participant attrition and was therefore judged to raise some concerns, with an unpredictable direction of bias because the influence of potentially missing data on the treatment estimate could not be determined. Although Schindl et al.^(10)^ reported two dropouts, the predicted direction of bias due to missing outcome data was judged to be toward the null.

### Certainty assessment

The GRADE framework evaluates certainty of evidence by considering risk of bias, inconsistency, indirectness, imprecision, publication bias, and other relevant factors. We evaluated three outcomes: reduction in recurrence frequency,^10^ recurrence severity,^(11,12)^ recurrence duration.^(11)^ Risk of bias was rated as serious due to one study ^(11)^ at high risk and another with some concerns,^(12)^ primarily related to blinding and attrition. Inconsistency was not rated as serious because all studies consistently favored photobiomodulation, and no meta-analysis was performed to calculate I^2^. Indirectness was rated as serious due to differences in comparators (placebo vs. acyclovir) and variation in laser parameters across studies. Imprecision was rated as serious because confidence intervals were not reported for any outcome, sample sizes were small (total n<100 for recurrence frequency), and meta-analysis was not feasible. Overall, the certainty of evidence was downgraded to low for all outcomes. The importance of outcomes (critical versus important) was determined based on clinical relevance, independent of certainty ratings. only recurrence frequency was rated as “critical” because this was the primary outcome table 5.

**Table 5:** Summary of findings according to GRADE: quality of evidence and effect of interventions on recurrence frequency

Certainty assessment						Impact	Certainty	Importance
Nº of studies	Study design	Risk of bias	Inconsistency	Indirectness	Imprecision			
Recurrence frequency (assessed with median recurrence free interval )								
3	randomised trials	serious^a^	not serious	serious^b^	serious^c^	May reduce recurrence frequency, but evidence is uncertain.	⨁⨁◯◯ Low^a,b,c^	CRITICAL
Recurrence severity (assessed with size of the lesions)								
2	randomised trials	serious^a^	not serious	serious^b^	serious^d^	May reduce lesion size, but evidence is uncertain.	⨁⨁◯◯ Low^a,b,d^	IMPORTANT
Recurrence duration (assessed with evaluation posttreatment)								
1	randomised trials	serious^a^	not serious	serious^b^	serious^e^	May reduce duration, but evidence is very uncertain.	⨁⨁◯◯ Low^a,b,e^	IMPORTANT

95% CI: confidence interval

Explanations

a. The ROB 2 assessment identified one study at high risk (Zanella et al., 2022) and another with some concerns (Carvalho et al., 2010). Limitations included inadequate blinding in placebocontrolled studies and incomplete attrition reporting. We therefore judged the body of evidence to have serious methodological limitations.

b. Regarding the comparator, two studies used placebo instead of the standard treatment. In Schindl et al (1999) and Zanella et al. (2022), a placebo was used (i.e., laser turned off), whereas Carvalho et al. (2010) used 5% Acyclovir in the Control Group. Moreover, the intervention was similar but not identical across studies, with variations in laser device, wavelength, energy density (J/cm^2^), energy per point, total energy, application area (cm^2^), application time, and application interval, among other parameters.

c. Imprecision was rated as serious because 95% confidence intervals (95%CIs) were not reported for any of the included studies, preventing a direct assessment of the range of plausible effect sizes. In addition, the total sample size across the three studies was small (n =92), falling below the optimal information size needed for a precise and stable estimate of the treatment effect. The lack of meta-analytic pooling due to clinical and methodological heterogeneity further limited our ability to quantitatively assess precision. Therefore, we cannot confidently exclude the possibility of clinically meaningful benefit or harm.

d. For the outcome “severity of recurrences,” imprecision was rated as serious because only two studies contributed data (Carvalho et al., 2010; Zanella et al., 2022), with a combined sample size of 72 participants. Confidence intervals were not reported, precluding an assessment of the precision of the effect estimates. The small sample size and absence of reported CIs mean that the true effect may be substantially different from the observed estimate, and we therefore judged the evidence to have serious imprecision.

e. For the outcome “duration of recurrences,” imprecision was rated as serious because only one study (Zanella et al., 2022) contributed data, with a sample size of 32 participants. Confidence intervals were not reported, and the single-study estimate does not provide sufficient precision to confidently determine the magnitude of the effect. Given the small sample and absence of 95%CIs, we judged the body of evidence to have serious imprecision.

Note on the “Importance” column: The classification of importance (critical versus important) reflects the priority of each outcome for clinical decisionmaking, not the certainty of the evidence. Therefore, despite the downgrade in certainty to Low, the importance ratings remain unchanged: CRITICAL for reduction in recurrence frequency (the primary outcome) and IMPORTANT for severity and duration of recurrences (secondary outcomes). This distinction is consistent with GRADE guidance, which separates the assessment of certainty from the assessment of outcome importance.

### Reduction in recurrence frequency

Evidence for this outcome came from three randomized trials. Indirectness was rated as serious because two studies, Schindl et al.^(10)^ and Zanella et al.,^(11)^ sed a placebo comparator (laser switched off), while one, Carvalho et al.,^(12)^ used 5% acyclovir as an active control. In addition, the photobiomodulation protocols varied considerably across studies in terms of device, wavelength, energy density, energy per point, total energy, irradiated area, application time, and interval between sessions. Indirectness was therefore rated as serious. Imprecision was rated as serious because no confidence intervals were reported, effect estimates were incompletely described, and the total sample size was small, precluding a precise estimate of the treatment effect.

### Recurrence severity: lesion size

Two studies^(11,12)^ contributed to this outcome. Indirectness was rated as serious for the same reasons described above (differing comparators and laser protocols). Imprecision was rated as serious because confidence intervals and detailed effect estimates were not reported, and the combined sample size was limited.

### Recurrence duration

Only one study contributed to this outcome.^(11)^ Indirectness was rated as serious due to variation in intervention parameters and comparators. Imprecision was rated as serious because confidence intervals were not reported, the sample size was small, and a single study provides limited precision for estimating the effect.

## DISCUSSION

This systematic review evaluated the evidence on photobiomodulation for preventing herpes labialis recurrences and identified limitations and research gaps in the available literature. The evidence base is limited by the small number of eligible studies and recurrent methodological shortcomings. These limitations reduce confidence in the reported effects and underscore the need for better-designed trials.

Two included studies reported lower recurrence frequencies after photobiomodulation than after placebo irradiation.^(10,11)^ Carvalho et al.^(12)^ found no statistically significant between-group difference in recurrence frequency. Zanella et al.^(11)^ and Carvalho et al.^(12)^ also reported smaller recurrent lesions at selected assessment points. Carvalho et al.^(12)^ additionally reported reduced inflammatory edema. However, methodological limitations and clinical heterogeneity reduce confidence in these findings. Accordingly, the findings should be interpreted cautiously and do not support definitive clinical recommendations. Further well-designed randomized controlled trials using standardized intervention protocols, comparators, and outcome measures are needed to clarify treatment effects and identify appropriate clinical protocols.

Several methodological limitations were identified across the included studies, including small sample sizes. None of the included studies reported an a priori sample-size calculation, which may reduce confidence in the reliability of the findings. Without an appropriate estimate of the required sample size, a study may be underpowered, increasing the risk of a type II error and limiting the precision and interpretability of its findings.

Two studies did not report participant attrition. Given the extended follow-up periods, the absence of reported attrition should not be interpreted as evidence that no participants withdrew. Attrition information was not reported by Zanella et al.^(11)^ or Carvalho et al.^(12)I^n both studies, outcome assessment and participant interviews were not blinded.^(11,12)^

Schindl et al.^(10)^ and Zanella et al.^(11)^ reported no adverse events, whereas Carvalho et al.^(12)^ did not provide adverse-event information. The limited available data did not identify treatment-related adverse events, but they are insufficient to establish safety conclusively. All included controlled trials enrolled adults; therefore, the effectiveness and safety of preventive photobiomodulation in children remain unknown. The following sections discuss key uncertainties regarding preventive treatment protocols.

### Optimal wavelength: red or near-infrared light

The wavelength most effective for preventing herpes labialis recurrences remains unclear. This remains an important gap in the literature. Among the included studies, Schindl et al.^(10)^ used visible red light at 690nm and reported a significant reduction in recurrence frequency. Zanella et al.^(11)^ and Carvalho et al.^(12)^ used near-infrared wavelengths of 808 and 780nm, respectively; one reported a significant preventive effect, whereas the other did not. The optimal target tissue for irradiation also remains uncertain. Visible red light penetrates relatively superficial tissues, whereas near-infrared light reaches deeper tissue layers.^(17)^ It is therefore unclear which wavelength is more likely to produce the greatest preventive benefit. Potential targets include epithelial tissues involved in local viral replication and deeper neural structures involved in viral transport from the sensory ganglion.^(1)^ The target associated with the greatest clinical benefit has not been established. It is also unknown whether one wavelength is superior or whether both provide clinically meaningful benefits. Combined use of red and near-infrared wavelengths has not been adequately evaluated. Any additional benefit from a combined protocol therefore remains uncertain. The available evidence does not resolve these questions. Both red and near-infrared protocols have shown potentially favorable effects in individual studies, but the evidence is too limited for direct comparison. Future trials should directly compare red and near-infrared wavelengths and evaluate whether combined-wavelength protocols offer additional benefit.

### Anatomical application site: usual recurrence sites or the entire perioral region

Herpes labialis recurrences may develop at several perioral sites, including the lips, surrounding skin, chin, and nasal region. The optimal anatomical distribution of preventive irradiation remains uncertain. It is unclear whether treatment should be restricted to the patient’s usual recurrence sites or extended across the entire perioral region. This question remains unresolved in the literature. Schindl et al.^(10)^ applied laser irradiation to the site of the patient’s original recurrent infection. When recurrences affected both the upper and lower lips, both sites were irradiated (Table 3). Carvalho et al.^(12)^ irradiated approximately 60 points across the lips and surrounding region, whereas Zanella et al.^(11)^ treated approximately 40–50 points throughout the labial and perioral areas (Table 3). Given the small number of studies and substantial variation in application sites, the optimal irradiation area requires further investigation.

### Optimal number and frequency of treatment sessions

All three included studies administered an initial course of 10 photobiomodulation sessions (Table 3).^(10-12)^ However, session frequency varied considerably, ranging from daily treatment in Schindl et al.^(10)^ to treatment every 48 hours in Zanella et al.^(11)^ and once weekly in Carvalho et al.^(12)^ Furthermore, Carvalho et al.^(12)^ found no statistically significant difference in recurrence frequency between the groups Because this study differed from the other trials in several respects, its findings cannot be attributed specifically to the once-weekly treatment schedule. Whether longer intervals between sessions reduce preventive effectiveness remains unknown. The available evidence does not establish whether shorter treatment intervals are superior. The optimal treatment frequency therefore remains an important knowledge gap.

### Optimal timing of preventive treatment initiation

The review included studies that administered preventive photobiomodulation while participants had no active lesions. Only three randomized clinical trials met these eligibility criteria. Two studies were excluded because photobiomodulation was initiated during active outbreaks, although both also assessed subsequent recurrence frequency and reported potentially favorable preventive outcomes.^(18,19)^These data are presented as background information and not as evidence derived from the studies included in the review. These findings raise the question of whether preventive effectiveness depends on initiating treatment during an asymptomatic period or whether similar long-term benefits may occur when treatment begins during an active episode.

The two excluded studies that initiated laser phototherapy during active recurrences and subsequently evaluated recurrence frequency were Vélez-González et al.^(18)^and Muñoz Sanchez et al.^(19)I^n a randomized double-blind clinical trial, Vélez-González et al.^(18)^ evaluated 36 patients older than 16 years with labial or facial herpes. Participants were assigned to three groups: oral acyclovir 200 mg five times daily for 10 days plus sham helium–neon laser treatment (n=10); helium–neon laser treatment plus placebo acyclovir (n=12); or combined helium–neon laser treatment and acyclovir (n=14). The helium–neon laser had a wavelength of 632.8nm, a power output of 20mW, a 2-mm-diameter optical fiber, an energy density of 8J/cm^2^. Laser irradiation was applied at several points within the affected area and adjacent tissue. The participants’ mean age was 26.5 years. Six helium–neon laser sessions were administered: the first two on consecutive days and the remaining four on alternate days. The duration of follow-up was not reported. The authors reported a significant between-group difference in annual recurrence frequency. Recurrences decreased from 7.83 to 1.16 episodes per year in the laser-only group and from 7.28 to 1.26 episodes per year in the combined-treatment group, compared with a decrease from 5.2 to 2.8 episodes per year in the acyclovir-only group. Healing time was shorter with combined laser treatment and acyclovir (7.85 days) than with laser treatment alone (9.16 days) or acyclovir alone (9.00 days).^(18)^ In the other study, Muñoz Sanchez et al.^(19)^ evaluated photobiomodulation in patients with recurrent herpes labialis. The author did not apply the treatment as a preventive intervention in the absence of symptoms. Instead, the treatment was administered during symptomatic episodes, which led to the exclusion of this study due to the predefined protocol criteria. The laser protocol used a wavelength of 670nm, noncontact application, a power output of 40mW, an irradiation time of 40s, an energy density of 2.04J/cm^2^, and a reported power density of 51mW/cm^2^. Energy delivery was 1.6J per lesion during the prodromal phase, 4.8J during the vesicular or secondarily infected stage, and 1.2J over the C2–C3 region. The comparator included topical or oral acyclovir or other palliative treatment, such as topical anesthetic therapy. The number of laser-phototherapy sessions was not reported. The study evaluated lesion-healing time. A total of 322 patients aged 18–59 years were monitored for 5 years to assess recurrence frequency. Participants were interviewed annually. The laser group reportedly experienced longer intervals between herpes labialis recurrences. No adverse events were reported. The study did not report a statistical analysis of recurrence-frequency outcomes. Among participants with monthly recurrences, the number of affected patients changed from 9 before treatment to 0 after treatment in the laser group and from 7 to 6 in the Control Group. For recurrences every 2–3 months, the corresponding counts changed from 26 to 0 and from 24 to 21; for recurrences every 4–5 months, they changed from 58 to 37 and from 56 to 46, respectively.

Future studies should determine whether preventive photobiomodulation must be initiated during an asymptomatic period or whether treatment begun during an active outbreak provides comparable long-term preventive effects.

During the preventive protocols of Zanella et al.^(11)^ and Carvalho et al.,^(12)^ some participants developed active lesions. These recurrences were treated using photodynamic therapy in Zanella et al.^(11)^ and a different laser parameter in Carvalho et al.^(12)^ These additional interventions represent a potential source of co-intervention bias and should be considered when interpreting recurrence outcomes.

### Therapeutic window: stimulatory or inhibitory dose

The three studies used similar low laser-power outputs of 60, 80, and 100mW; however, energy densities varied substantially, from 3 and 4.5J/cm^2^ in Carvalho et al.^(12)^ to 11.11 and 22.22J/cm^2^ in Zanella et al.^(11)^ and 48J/cm^2^ in Schindl et al.^(10)^ (Table 3). Given this wide variation in energy density, the optimal dosing range remains uncertain, including whether preventive protocols should target stimulatory effects in host tissues or inhibitory effects on viral activity. Mester’s therapeutic-window concept^(20)^ proposes that low-level laser therapy produces beneficial biological effects, including tissue repair, reduced inflammation, and pain relief, within an appropriate dose range. Doses below this range may be ineffective, whereas excessive energy may inhibit cellular activity or cause tissue damage. This concept emphasizes precise dosimetry, with stimulatory effects reported at energy densities of approximately 0.05–10J/cm^2^.^(20)^ Future studies should determine the energy density and total delivered dose that provide the most favorable preventive effect.

### Limitations of the review process

A key limitation of the included studies was the lack of clarity regarding participants’ awareness of their assigned interventions. In Schindl et al.^(10)^ and Zanella et al.,^(11)^ it was unclear whether sham procedures adequately prevented participants from identifying their treatment allocation. Inadequate blinding may have introduced performance bias because participant expectations could influence self-reported outcomes. Similarly, Carvalho et al.^(12)^ compared visibly different treatment protocols but did not clarify what participants were told about the alternative intervention. This limited reporting makes it difficult to determine whether expectations or knowledge of treatment assignment influenced adherence, reporting, or perceived effectiveness.

Substantial heterogeneity among the studies also limited direct comparison. This heterogeneity arose from wide variation in laser parameters, treatment schedules, comparators, outcome definitions, and follow-up procedures, together with the small number of eligible randomized trials. These limitations underscore the need for additional randomized controlled trials of photobiomodulation for preventing recurrent herpes labialis.

The following sections summarize methodological limitations of the included studies, unresolved research questions, and reporting elements needed to improve protocol reproducibility and comparability across future trials.

### Methodological limitations of the included studies

Several recurring methodological limitations should be addressed in future clinical trials. None of the studies reported an a priori sample-size calculation, raising the possibility of inadequate statistical power and type II error. Although all studies were described as randomized, none reported the method used to generate the allocation sequence, limiting assessment of selection bias. Blinding procedures were also inconsistent: one study was double-blind, one was single-blind, and one was unblinded. Another methodological concern involved the analysis of baseline recurrence frequency. The studies generally analyzed participants as a single group using aggregate recurrence values rather than stratifying them by baseline recurrence frequency. This approach may have obscured differences in treatment response between participants with lower and higher baseline disease burden. The placebo-controlled studies also did not report whether inactive laser devices reproduced operational cues, such as the activation sound. Adequate simulation of the active procedure is important for maintaining participant blinding and reducing expectation-related bias. In Zanella et al.,^(11)^ and Carvalho et al.^(12)^ studies, the same clinician administered the intervention and assessed clinical outcomes, increasing the potential for detection bias.

### Incomplete reporting of laser protocols

Another important limitation was incomplete reporting of laser-irradiation protocols, which reduced reproducibility and limited meaningful comparisons across trials. Future studies should report all relevant photobiomodulation parameters, including the laser device and manufacturer, country of origin, wavelength, operating mode, application mode, energy density, energy delivered per point, total energy, irradiated area, number and anatomical location of irradiation points, spot size, sham-irradiation procedures, power output, power density, and verification of power output with a calibrated power meter. Treatment-related variables should also be reported consistently, including irradiation time per point, interval between sessions, total number of sessions, overall treatment duration, and follow-up duration. Future studies should clearly describe the frequency and method of recurrence assessment throughout follow-up and report recurrence outcomes using standardized measures, preferably as episodes per month or per person-time unit. Standardized and complete reporting is essential for protocol reproducibility, cross-study comparison, and future quantitative synthesis.

### Priorities for future research

Despite potentially favorable findings, several clinical and methodological questions remain unresolved. Future randomized trials should directly compare red and near-infrared irradiation to determine whether either wavelength provides superior preventive outcomes. The optimal time to initiate preventive photobiomodulation also remains unclear, including whether treatment should begin during an asymptomatic period or whether initiation during an active outbreak affects long-term efficacy. The number and frequency of sessions required to achieve a sustained preventive effect have not been established. Further research should determine whether irradiation should be restricted to each patient’s usual recurrence sites or extended across the entire perioral region. The optimal therapeutic window also remains uncertain, including the dose range most likely to produce a clinically meaningful preventive effect. Addressing these questions through well-designed randomized controlled trials will be necessary to establish standardized, evidence-based photobiomodulation protocols for preventing herpes labialis recurrences.

## CONCLUSION

The available evidence suggests a potential preventive benefit but remains insufficient to support definitive clinical recommendations. The included studies reported some favorable outcomes, and the studies that provided safety information reported no adverse events. However, the certainty of this evidence is low. Confidence in the findings is limited by small sample sizes, absent sample-size calculations, incomplete blinding, and substantial heterogeneity in laser parameters (e.g., wavelength, energy density, application time) and comparator interventions. These methodological and clinical differences restrict generalizability and prevent the formulation of evidence-based clinical recommendations. Well-designed randomized controlled trials using standardized laser parameters, clearly defined comparators, and consistent outcome measures are needed to establish the optimal preventive protocol and confirm the clinical utility of photobiomodulation for recurrent herpes labialis.

## SUPPLEMENTARY MATERIAL

Supplementary material 1
