# Supplementary material for: Can photobiomodulation prevent herpes labialis recurrences? A systematic review
Source: Einstein (Sao Paulo). 2026 Sep 1;24(spe3):eRW2359. doi: 10.31744/einstein_journal/2026RW2359 (PMC13689959; doi:10.31744/einstein_journal/2026RW2359)
Supplement: Supplementary file 1 [file 2317-6385-eins-24-nspe3-eRW2359-suppl01.pdf]

SUPPLEMENTARY MATERIAL

Can photobiomodulation prevent herpes labialis recurrences? A systematic review

Karen Müller Ramalho, Nicolly Dias dos Santos, Juliana Mota Siqueira, Anna Carolina Ratto Tempestini Horliana, Carlos de Paula Eduardo, Leticia Mello Bezinelli, Fernanda de Paula Eduardo, Anna Luíza Damaceno Araújo

DOI: 10.31744/einstein_journal/2026RW2359

Table 1S. Database-specific search strategies and numbers of records retrieved

Database or source	Search strategy<br>Date of final search: July 20, 2026	Records retrieved, n	Subtotal, n
Cochrane Library	(photobiomodulation OR "low-level laser therapy") AND (herpes OR "herpes simplex" OR "herpes labialis" OR HSV)	18	Database subtotal: 798
Embase	('photobiomodulation'/exp OR photobiomodulation OR 'low level laser therapy'/exp OR 'low level laser therapy') AND (herpes OR 'herpes simplex' OR 'herpes labialis')	289	
Regional Portal of the Virtual Health Library*	(photobiomodulation OR fotobiomodulação OR fotobiomodulación OR "low-level laser therapy" OR "laser de baixa potência" OR "láser de baja potencia") AND (herpes OR "herpes simplex" OR "herpes simples" OR "herpes labialis" OR "herpes labial" OR HSV)	119	
PubMed	(photobiomodulation OR "low-level laser therapy") AND (herpes OR "herpes simplex" OR "herpes labialis")	76	
Scopus	TITLE-ABS-KEY((photobiomodulation OR "low-level laser therapy") AND (herpes OR "herpes simplex" OR "herpes labialis"))	296	
Google Scholar	(photobiomodulation OR "low-level laser therapy") AND (herpes OR "herpes simplex" OR "herpes labialis" OR HSV)	100	Gray literature subtotal: 140
ProQuest	NOFT(photobiomodulation OR "low-level laser therapy") AND NOFT(herpes OR "herpes simplex" OR "herpes labialis" OR HSV)	40	
Total		Total records retrieved: 938	

*The Regional Portal of the Virtual Health Library includes BBO: *Bibliografia Brasileira de Odontologia*; BDENF: *Base de Dados de Enfermagem*; BINACIS: *Biblioteca Nacional de Ciências da Saúde, Argentina*; CUMED: *Base de Dados de Literatura de Ciências da Saúde de Cuba*; IBECS: *Índice Bibliográfico Espanhol de Ciências da Saúde*; LILACS: *Literatura Latino-Americana e do Caribe em Ciências da Saúde*; LIPECS: *Literatura Peruana de Ciências da Saúde*.

Table 2S. Reports excluded after full-text assessment and reasons for exclusion

n	Author	Title	Year	Country	Journal or conference	Reason for exclusion
1	Alster et al.	"Dermatologic laser side effects and complications: prevention and management."	2020	United States	American Journal of Clinical Dermatology	Reason for exclusion: Review article
2	Ferreira et al.	"Recurrent herpes simplex infections: laser therapy as a potential tool for long-term successful treatment."	2011	Brazil	Revista da Sociedade Brasileira de Medicina Tropical	Reason for exclusion: Case report
3	Ferreira et al.	"Impact of low-intensity laser on the suppression of infections caused by herpes simplex viruses 1 and 2: an *in vitro* study."	2009	Brazil	Revista da Sociedade Brasileira de Medicina Tropical	Reason for exclusion: *In vitro* study
4	Namvar et al.	"Effect of photodynamic therapy using 810- and 940-nm diode lasers on herpes simplex virus type 1: an *in vitro* study."	2019	Iran	Photodiagnosis and Photodynamic Therapy	Reason for exclusion: *In vitro* study
5	de Paula Eduardo et al.	"Prevention of recurrent herpes labialis outbreaks through low-intensity laser therapy: a clinical protocol with 3-year follow-up."	2012	Brazil	Lasers in Medical Science	Reason for exclusion: Uncontrolled prospective case series
6	Seyyed et al.	"Efficacy of adjuvant photobiomodulation therapy in recurrent herpes labialis: a randomized clinical trial."	2024	Iran		Reason for exclusion: Photobiomodulation was used as adjunctive treatment rather than as a preventive intervention
7	Al-Hallak et al.	"Efficacy of photobiomodulation therapy in recurrent herpes labialis management: a randomized controlled trial."	2024	Syria	Clinical Oral Investigations	Reason for exclusion: Photobiomodulation was not used as a preventive intervention
